# Cell-free DNA analysis in current cancer clinical trials: a review

**DOI:** 10.1038/s41416-021-01696-0

**Published:** 2022-01-13

**Authors:** M. Cisneros-Villanueva, L. Hidalgo-Pérez, M. Rios-Romero, A. Cedro-Tanda, C. A. Ruiz-Villavicencio, K. Page, R. Hastings, D. Fernandez-Garcia, R. Allsopp, M. A. Fonseca-Montaño, S. Jimenez-Morales, V. Padilla-Palma, J. A. Shaw, A. Hidalgo-Miranda

**Affiliations:** 1 Laboratorio de Genómica del Cáncer, Instituto Nacional de Medicina Genómica, Mexico, Periférico Sur No. 4809, Col. Arenal Tepepan, Delegación Tlalpan, Ciudad de Mexico, 14610 Mexico City, Mexico; 2 Leicester Cancer Research Centre, Department of Genetics and Genome Biology, University of Leicester, University Road, Leicester, LE1 7RH UK

**Keywords:** Cancer, Tumour biomarkers, Predictive markers

## Abstract

Cell-free DNA (cfDNA) analysis represents a promising method for the diagnosis, treatment selection and clinical follow-up of cancer patients. Although its general methodological feasibility and usefulness has been demonstrated, several issues related to standardisation and technical validation must be addressed for its routine clinical application in cancer. In this regard, most cfDNA clinical applications are still limited to clinical trials, proving its value in several settings. In this paper, we review the current clinical trials involving cfDNA/ctDNA analysis and highlight those where it has been useful for patient stratification, treatment follow-up or development of novel approaches for early diagnosis. Our query included clinical trials, including the terms ‘cfDNA’, ‘ctDNA’, ‘liquid biopsy’ AND ‘cancer OR neoplasm’ in the FDA and EMA public databases. We identified 1370 clinical trials (FDA = 1129, EMA = 241) involving liquid-biopsy analysis in cancer. These clinical trials show promising results for the early detection of cancer and confirm cfDNA as a tool for real-time monitoring of acquired therapy resistance, accurate disease-progression surveillance and improvement of treatment, situations that result in a better quality of life and extended overall survival for cancer patients.

## Background

Circulating cell-free DNA (cfDNA) and the tumour-derived DNA fraction, circulating tumour DNA (ctDNA), can be analysed in the context of a liquid biopsy (LB). Analysis of cfDNA is becoming an important aid in the prognosis, identification of specific genomic alterations, selection of targeted therapies and to provide information regarding treatment efficacy over time [1]. The feasibility to detect tumour-specific genomic alterations in cfDNA obtained from the plasma of cancer patients has been well documented [2, 3]. Several methods have been used to quantify and detect tumour mutations in cfDNA/ctDNA. The foremost includes PCR, next-generation sequencing (NGS)-based approaches (i.e. whole-exome or whole-genome sequencing), cancer personalised profiling by deep sequencing (CAPP-Seq) and tagged-amplicon deep sequencing (TAm-Seq) [4–12]. These technologies and applications have been reviewed elsewhere [13]. However, for the routine clinical application of ctDNA/cfDNA analysis, the standardisation of pre- and post analysis must be further developed. Several studies have highlighted the significance of rigorous pre-analytical conditions to assess variability and integrity between samples to ensure high-quality molecular tests [14–19]. Even though ctDNA assays have clinical validity and utility in specific tumours in advanced stages, for the majority of the current assays, there is still not sufficient evidence to prove their clinical validity and utility, either in advanced tumours, in early stages of the disease or as a cancer-screening tool [20, 21]. Nowadays, the field is advancing rapidly and data generated by clinical trials using these analyses will be fundamental to determine its effectiveness as a cancer-screening tool and monitoring.

**Fig. 1:**
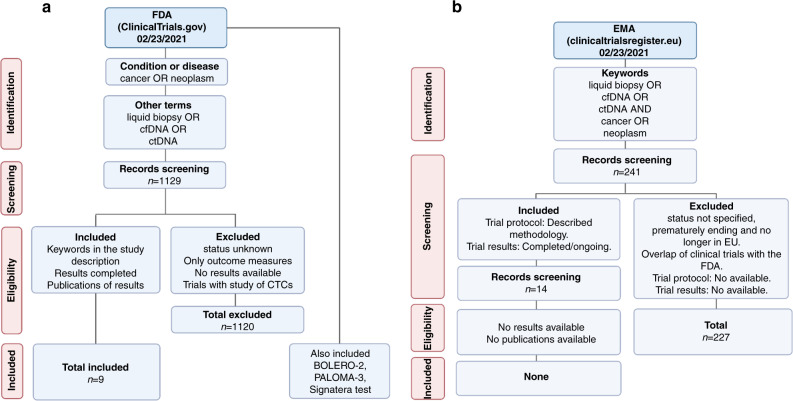
PRISMA diagram of the query strategy. **a** The diagram of FDA clinical trials, **b** the diagram of the EMA trials.

Given the current relevance and its potential clinical impact in the near future, in this review, we analysed clinical trials, including cfDNA OR ctDNA OR LB analysis as part of their main objective, to explore the current landscape of its applications, the tumour types where it is being used the most, the type of technologies used in clinical trials and its potential to improve not only advanced cancer clinical care and treatment monitoring, but also its potential role as a screening and early cancer-detection tool. To investigate this, an exhaustive search of clinical trials involving the study of LB in any type of cancer was carried out in the international public databases ClinicalTrials.gov of the Food and Drug Administration (FDA) (https://clinicaltrials.gov/) and in the European Union Clinical Trials Registry of the European Medicines Agency (EMA) (https://www.clinicaltrialsregister.eu/ctr-search/search). We obtained the screening records and downloaded them in binary file format (.xls). Then, we filtered the clinical trials based on “completed”, “recruited” or “ongoing” status and searched for their respective NCT or EudraCT Number at the FDA or EMA databases. We selected the trials according to their relevance in the clinical setting. Then, we excluded trials with unavailable results or unknown status. The query strategy and the selected trials are described in Fig. 1a and b.

## Current use of liquid biopsies in cancer-related clinical trials

**Fig. 2:**
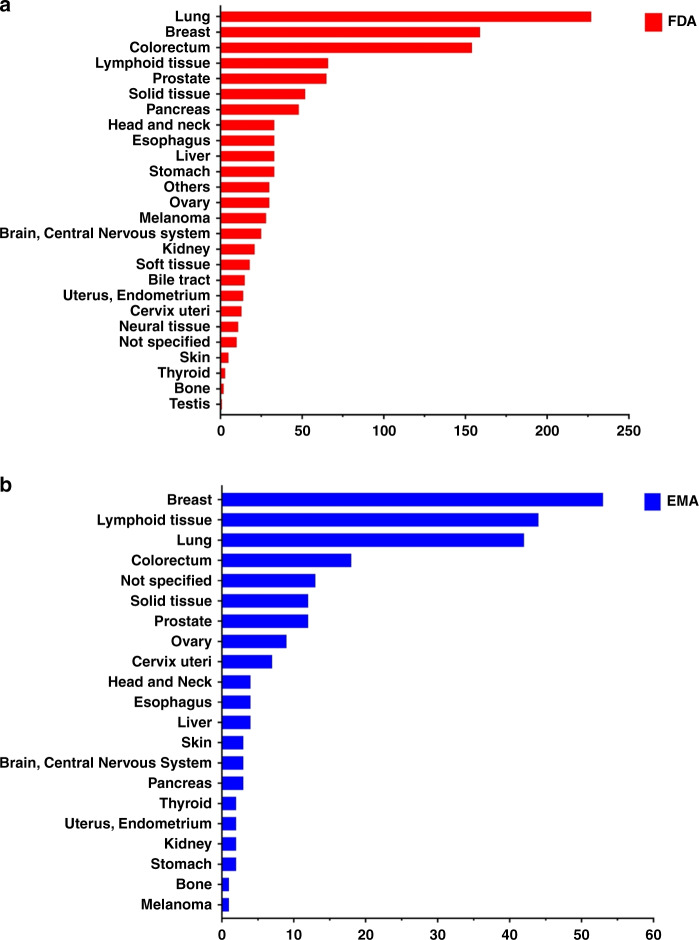
Clinical trials involving cfDNA/ctDNA analysis in different types of cancer. **a** Clinical trials found in the FDA public database (https://clinicaltrials.gov/). **b** Clinical trials identified in EMA (https://www.ema.europa.eu/en/human-regulatory/research-development/clinical-trials-human-medicines) public database.

A thorough search for clinical trials related to LB in cancer, on ClinicalTrials.gov (FDA) and EMA, identified 1129 and 241 clinical trials, respectively (date of query: 2021-02-25). In both queries, we observed that the tumours with the highest number of clinical trials were the most frequent in incidence and mortality such as lung, breast and colon cancer (Fig. 2a and b) [22]. It is worth mentioning that some clinical trial entries did not specify tumour type (Fig. 2b), however, we considered them because of the usefulness of LB analysis for the evaluation of cancer progression (Fig. 2a).

**Fig. 3:**
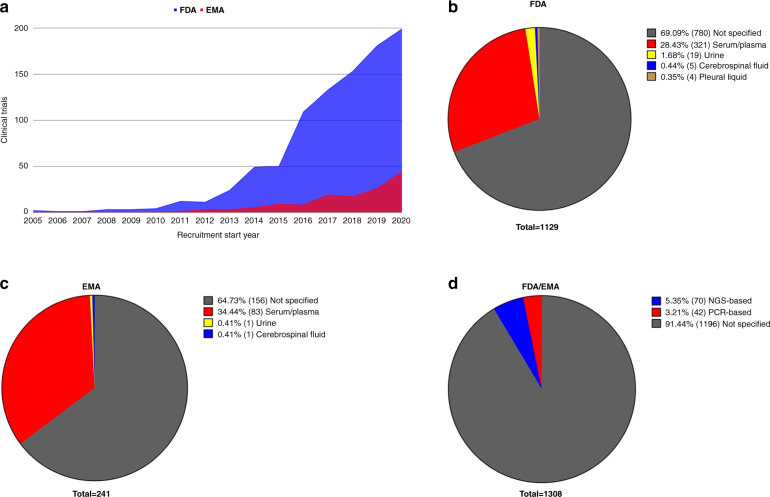
Number of clinical trials involving cfDNA analysis since 2005 and type of sample used for the analysis. **a** The number of clinical trials including cfDNA analysis in the last 15 years. **b**, **c** The type of sample analysed in FDA and EMA clinical trials, respectively. **d** The type of technology used for cfDNA analysis in both the FDA/EMA clinical trials included in the review.

cfDNA/ctDNA analysis can be performed using different biological samples. Based on the results of our query, blood samples (serum or plasma) were the main source of cfDNA/ctDNA, followed by urine, cerebrospinal fluid and pleural fluid samples (Fig. 3b and c).

The screening methods for the analysis of molecules in LB are based on NGS or specific methods based on PCR [23, 24]: both technologies are applied in the clinical trials in our query. It should be noted that most of the clinical trials do not mention the technology they used, possibly because they are phase- I or -II trials and the technology to be used has not yet been determined (Fig. 3d).

Most of the clinical trials (in this review 1370 studies) are based on the analysis of molecules that are released in different body fluids, falling into the ‘liquid biopsies’ term. These studies are focused on evaluating relevant information on the efficacy of new chemotherapeutic drugs, monoclonal antibodies, novel treatment combinations or patient follow-up, through the evaluation of risk of relapse (RR), minimal residual disease (MRD), overall survival (OS), disease-free survival (DFS) and effect of physical activity on cancer patients, among others.

### Biological and technical challenges in the liquid-biopsy field

We detected some omissions and limitations in the clinical trial information entries. Regarding the FDA trials, there were few completed (*n* = 185/1129, 16.4%) and from these, only 25 (2.2%) reported results. There are no results for 159 completed trials (14%) (start year min/max 2004/2020, end year min/max 2007/2020). Similarly, there is no information available for the remaining trials (*n* = 944, 83.6%; start min/max 2000/2020 and finish min/max 2011/2033). Therefore, it is challenging to evaluate the clinical relevance of LB for cfDNA/ctDNA analysis and its early adoption for routine usage in clinical practice.

As mentioned, LB tests are not yet fully implemented in routine cancer care, as there are still biological and technical hurdles. Currently, the FDA has approved five LB tests: FoundationOne Liquid CDx [25], Guardant360 CDx [26], COBAS EGFR mutation test V2 [27], Therascreen PIK3CA RGQ PCR [28] and Epi proColon, for SETP9 methylation detection in plasma [29].

In this section, we describe some of the main drawbacks observed across the selected trials.

Lack of consensus on terminology. There are terms that explain the structural and biological nature of cfDNA. Most of them are equivalent or contradictory. Even though there is no immediate term conflict, this could pose limitations between interstudy correlations and be a source of bias. To address this issue, Bronkhorst et al. described a thorough nomenclature for LB [30].

Unknown cfDNA origin. Tumoral and tumour microenvironment-associated cells release two types of DNA into the extracellular space: DNA that harbours cancer driver mutations and non-cancer DNA [31]. These non-cancer fragments also feed the cfDNA pool. Their contributions to the cfDNA pool fluctuate between cancer stages [3]. Furthermore, it has been reported that normal cells can harbour tumour-related mutations [32, 33]. These factors can increase the false-positive rate of the analysis [34].

To address this issue, multiple cancer-associated mutations can be screened simultaneously, as this can improve the probability of detecting ctDNA [35, 36]. Moreover, integration of multiple biological levels of information, such as DNA methylation and mutation status, can increase specificity of the analyses.

DNA release mechanisms and clearance rates. cfDNA levels are determined by the rate at which they are cleared from the blood, approximately 16 min to 2.5 h [37, 38]. The half-life of cfDNA varies among patients due to several biological and physiological factors [39, 40]. The clearance rates must be standardised and be validated in larger and independent cohorts. The mechanisms of the cfDNA clearance have yet to be addressed in these studies.

Non-standardised protocols. Currently, there is no gold-standard method to assess cfDNA. The lack of standardised protocols is one of the hurdles hampering the application of cfDNA analysis in routine clinical laboratories. As such, improving the pre-analytical steps to recover high-quality cfDNA and increasing the sensitivity of the tests is fundamental [41]. This effort is fundamental to develop a standardised consensus guideline for LB tests and more multicentred trials must be conducted to achieve high-quality tests.

Clinical trial design. Most of the trials analysed have key aspects that can be improved. First, 64 of 1129 FDA trials (5.7%) were driven in small cohort sizes, median = 100 (range 1–99,481). Whereas 92.1% have cohort sizes less than 1000 patients. Only 88 of 1129 trials have larger cohorts. Patient cohort size is essential when validating novel methodologies for diagnosis, prediction and prognosis [42]. These trials should consider increasing the enrollment for statistical power purposes, especially for low-prevalence cancers.

An important consideration is the follow-up period, which could represent a significant challenge for the analysis of cfDNA/ctDNA, especially in clinical trials intended to identify tumours that have not yet been clinically detected or that remain inactive after the therapy. Monitoring clinical and biological changes associated with tumour development and tumour relapse for a long time might skew the information with the appearance of other degenerative diseases or uncontrolled drug consumption.

Despite these challenges, we select the most relevant clinical trials registered within the most important regulatory agencies in America and Europe (FDA and EMA), that include cfDNA/ctDNA analysis in high-frequency tumours such as lung, breast and colon cancers, as well as poor-prognosis tumours such as pancreatic cancer. These clinical trials reveal new hopes for the early detection of cancer, real-time monitoring of acquired therapy-resistance dynamics, more accurate disease-progression surveillance and improved treatment protocols that lengthen the OS for cancer patients. In the final section, we describe the importance of these trials and potential further directions in the field.

### Breast cancer: monitoring genes related with acquired treatment resistance

During antineoplastic treatment, breast cancer (BRCA) acquires mutations in the oestrogen-receptor gene (*ESR1*), one of the multiple mechanisms underlying resistance to aromatase inhibitor (AI) treatment [43]. An example of this effect is the L536H mutation, which was identified in ctDNA after AI therapies in patients with metastatic BRCA. Experimental studies showed that this mutation results in ligand-independent activation of the ER protein [44]. Under this context, the cfDNA or ctDNA analysis offers the opportunity to study therapy-resistance-related mutations in BRCA patients, which in turn might result in better patient care and extended life expectancy.

The BOLERO-2 clinical trial (NCT00863655) is a double-blind, phase-III, multicentre trial that assessed the prevalence of *ESR1* mutations (Y537S and D538G) by digital droplet PCR (ddPCR) in ctDNA from 541 metastatic BRCA patients treated with exemestane combined with everolimus or a placebo. The authors related both mutations with a reduced OS, in contrast with wild-type *ESR1* patients. Moreover, a shortened progression-free survival (PFS) is associated with D538G mutations, in contrast with wild-type *ESR1* patients. These results suggest that *ESR1* mutations emerge in ctDNA from metastatic BRCA patients with prior AI treatment, which can be detected by ddPCR, their presence is related with more aggressive tumours, and might be used as biomarkers for predicting outcome [45]. In addition, the BOLERO-2 trial explored the overall concordance of PIK3CA hotspot mutations (H1047R, E545K and E542K) present in ctDNA with hormone receptor positive (HR+) and HER2 negative (HER2−) in BRCA patients. The study found that the PIK3CA mutational status between archival tumour and cfDNA sample pairs was 70.4%, with a higher concordance (81.6%) for metastatic lesions [46]. These data show that, if both tumour material and plasma are collected simultaneously and the material is analysed using the same highly sensitive methodology, concordance between the two sample types is high [47].

Another approach was taken within the PALOMA-3 clinical trial (NCT01942135). Although the main objective of this clinical trial was to demonstrate the superiority of palbociclib in combination with fulvestrant (Faslodex) over fulvestrant alone to prolong PFS in women with HR+, HER2− metastatic BRCA, with disease progression after a previous endocrine therapy [48–50]. The authors aimed to evaluate *PIK3CA* and *ESR1* ctDNA mutations before, during and after treatment with a CDK4/6 inhibitor in the same group of patients. A combination of digital droplet PCR, targeted sequencing and whole-exome sequencing (WES) showed that more mutations were detected at the end of treatment compared with initial samples, with at least one new detectable/acquired mutation at the end of treatment (30.8%). There were also patients in both treatment groups who acquired at least one new mutation, (30.7%) in the palbociclib plus fulvestrant arm and (30.9%) in the placebo plus fulvestrant arm. Other patients receiving palbociclib plus fulvestrant acquired detectable *RB1* gene mutations at the end of treatment. In two cases, they had two *RB1* aberrations, which had been previously identified from WES, suggesting resistant polyclonal subclones. These observations support the appearance of *RB1* aberrations acquired or selected under palbociclib pressure, but only in a minority of patients [51]. Recent updates from the PALOMA-3 trial have also shown that patients with a high fraction of ctDNA have an overall worse PFS [52]. These data show that cfDNA analysis might facilitate clinical decisions for patients with treatment-refractory BRCA.

The CICLADES trial (NCT03318263), proposes to monitor cfDNA for mutations in *ESR1*, *PIK3CA* and *AKT* genes, key molecules in oestrogen-receptor-independent activation and its relationship with AI treatment resistance [53, 54]. This trial enrolled 146 participants to determine the prevalence of mutations in these genes and allow the early detection of resistance to AIs, as reported during the 2016 meeting of the American Society of Clinical Oncology (ASCO). The authors considered samples collected at the beginning of the study, in the progression point and 3 months before the progression. Although no results have been reported to date, we included this trial because few studies focus on preventing resistance to first-line treatments with cfDNA analysis. This topic has a relevant impact in BRCA clinical behaviour, as highly mutational changes in actionable genes can lead to resistant clones and subclones.

### Lung cancer: early detection and treatment monitoring

Most lung-cancer (LC) patients are diagnosed until they present symptoms, usually in advanced stages where the odds of a curative treatment are limited. With this goal in sight, the MILD trial was launched (NCT02837809). It was a prospective randomised controlled study (*n* = 4099; aged 49–75 years and smoking history within ten years). This trial combined smoking cessation with early diagnosis and biological assessment of the individual risk of LC. The intention was to measure the levels of cfDNA as a biomarker to assess whether this analysis can identify individuals at higher risk of cancer, improve the sensitivity and specificity of imaging techniques or both. The results showed that high levels of cfDNA are strongly associated with the presence of lung cancer, regardless of the stage [55–57]. Surprisingly, in small lung cancers, the ctDNA level was not able to discriminate between the healthy control or patients with other tumours. CtDNA levels were established as a poor-prognosis indicator for survival and correlated with disease aggressiveness. Furthermore, ctDNA levels tended to be much higher at both baseline and surgery for tumours in Stage II–IV. These data indicate that ctDNA levels are useful to determine prognosis in lung cancer, regardless of the stage of the disease.

One of the major issues in LC is treatment-response monitoring. For this reason, the BENEFIT trial (NCT02282267) aimed to validate gefitinib response in lung adenocarcinomas bearing *EGFR* mutations [58]. The trial included 339 LC tissue samples paired with blood samples to search for exon 19 (E746–A750) and/or exon 21 (Leu858Arg) *EGFR* mutations, which can adequately predict gefitinib-therapy response [59]. The results indicate that of 188 patients who received treatment with gefitinib and with *EGFR* mutations in ctDNA, 180 had *EGFR* mutations in both tissue and ctDNA and eight had *EGFR* mutations only in ctDNA. The ctDNA versus the tissue showed a high specificity of 93.9% and a positive predictive value of 95.8% for the *EGFR* mutation status, with a sensitivity of 70%. Furthermore, a high concordance for Thr790Met de novo was obtained between the reference tissue and plasma samples. This alteration is considered the most frequently reported acquired resistance mechanism in response to EGFR-TKI therapy [60]. Consequently, the authors suggest that the analysis of *EGFR* mutation dynamics through ctDNA examination can identify patients that would have no real benefit from EGFR-TKI treatment [61].

The clinical study NCT03059641 aimed to evaluate the concordance of clonal mutations in ctDNA, using a 1021 gene-targeted panel in plasma coupled with tumour tissue from patients with advanced-stage (IIB or IV) non-small-cell LC. In total, 72 patients harboured the *EGFR* gene dominant clone and multivariate analysis demonstrated that dominant *EGFR* clones are an independent prognostic indicator of the efficacy of first-line treatment with EGFR-TKI [62]. Importantly, genomic information from both tissue and ctDNA provides a better landscape of new and existing actionable mutations that could benefit patients from other therapeutic targets that could improve disease prognosis.

## Colorectal cancer: the importance of early detection

Efforts have been made to improve the early detection rate of colorectal cancer (CRC), as the 5-year survival rate is >80% in early stages, but decreases to <10% for late diagnosis of metastasised cancer [63]. AI-EMERGE (NCT03688906) is the first in Freenome’s AI-PATTERNS clinical trial series. This series focused on the development of a non-invasive blood test for the early detection of cancer. AI-EMERGE was the largest retrospective, international, multicentre, retrospective trial to date that analyses cfDNA in samples from patients with early-stage CRC. AI-EMERGE implemented automated machine learning (ML) to find and learn associations between cfDNA profiles and cancer ‘status’ to detect early-stage CRC. This study evaluated a total of 817 plasma samples, including 271 control samples and 546 CRC patient samples, of which 81% had early-stage cancer (stages I and II). The study performed whole-genome sequencing (WGS) on cfDNA and other multi-omics technologies to analyse other biomarkers in blood. The aim was to evaluate the sensitivity and specificity of their test between CRC stages and compare them with current standard screening methods. In the study, they demonstrated that ML-based analysis is useful to identify the relationship between a patient’s cfDNA profile and cancer diagnosis, with 85% sensitivity and 85% specificity in CRC using standard *k-fold* cross-validation. Further validation of the results and extension of this work to other cancers is currently underway [64]. With these results, although not entirely conclusive, the authors propose an alternative for the timely detection of CRC using non-invasive methods.

The ECLIPSE clinical trial (NCT04136002) is a prospective study to evaluate the performance of the LUNAR test in subjects with average risk for CRC. Previously, the authors have developed a methodology to identify genome and epigenome alterations in a single assay (LUNAR test) using plasma samples. This test incorporates somatic genomic variant detection, epigenomic analysis and a bioinformatic classifier to filter non-tumour variants. In this pilot study, ctDNA detection in early-stage CRC patients (I–III) had 94% specificity and incorporation of epigenomic analysis significantly enhanced ctDNA detection relative to somatic mutational analysis alone [65].

These studies reflect the utility of ctDNA testing as a non-invasive option for early detection of CRC, which can have a relevant impact in CRC incidence and mortality rates in the future.

## CfDNA-based clinical trials for poor-prognosis tumours

Although the majority of the clinical trials focus on the three most frequent tumours worldwide (see Fig. 2), it is important to describe the clinical trials that include ctDNA analysis in tumours with poor prognosis, such as pancreatic cancer, which is considered a lethal entity with poor outcomes and an increasing incidence [66].

An example is the Metformin001 clinical trial (NCT02978547), which has as a primary goal to evaluate metformin effects as a neoadjuvant for tumour progression as well as cell proliferation in pancreatic ductal adenocarcinoma (PDAC). In this study, the authors collected and stocked fresh tumour tissue, normal tissue, serum and plasma. Investigators searched for ctDNA mutations on *KRAS* by ddPCR, as it has been reported that *KRAS* is mutated with 96% frequency in PDAC [67, 68]. Mutations were detected in 74.7% of the tissue samples, while in ctDNA samples, the *KRAS* mutation rate was 64.6%. The concordance of the *KRAS* mutations between tissue DNA and ctDNA was 73% (58/75). Furthermore, the authors found that patients harbouring ctDNA *KRAS* G12V mutation presented significantly decreased survival rate (median = 276 days), compared with patients with wild-type *KRAS* (median = 413 days). These results suggest that the presence of G12V mutations in ctDNA has clinical utility as a prognostic tool and support the use of non-invasive techniques for the detection of mutations in patients with PDAC [69]. However, although there is a high concordance between KRAS in tissue samples and ctDNA, it is important to validate these findings in larger cohorts to assess the reproducibility of the data and have greater certainty in therapeutic decision-making.

Assessment of MRD in recurrent tumours is another use for ctDNA analysis. In a clinical case study, a patient experienced oligometastatic recurrence, despite aggressive trimodal therapy. At the time of oesophageal adenocarcinoma recurrence, elevated ctDNA levels were revealed, which after six cycles of treatment decreased significantly. However, during follow-up, ctDNA levels started to increase, while CEA (carcinoembryonic antigen) levels (a disease biomarker) decreased. One year later, the patient developed a new FDG (fluorodeoxyglucose) tumour mass in the ablation cavity, accompanied by an increase in the CEA level. The authors noted that ctDNA levels began to increase 174 days before the CEA increase and 350 days before radiographic detection of recurrence. After metastasectomy, surveillance imaging showed no evidence of recurrence, ctDNA levels became negative and the patient currently remains in remission (11 months after metastasectomy) [70]. It is clear that detectable ctDNA could help clarify ambiguous findings generated by the use of nonspecific biomarkers and identify patients at high risk of MRD who could be candidates for experimental adjuvant therapies.

## cfDNA analysis for early cancer detection: the Circulating Cell-free Genome Atlas Study (CCGA) and the STRIVE trials

These two studies are being conducted by GRAIL, Inc., which combines ultra-deep sequencing of cfDNA with ML algorithms to identify early cancer.

The CCGA trial (NCT02889978) started in 2016, collecting samples from cancer patients and subjects with not-known history or diagnosis of cancer. The analysed data were used to develop models capable of discriminating between cancer and non-cancer patients, as well as the tumour tissue of origin on ctDNA analysis. To determine the concordance between the primary and the ctDNA, they sequenced a tumour biopsy. This trial is an observational and prospective study (*n* = 15,000).

The STRIVE trial (NCT03085888) started in 2017, with the initial objective of establishing a prospective cohort of 120,000 women undergoing screening mammography that would be used to train and clinically validate a test for BRCA detection [71]. In 2019, the objective of the trial was expanded to include the diagnosis of invasive cancer, including haematologic cancer (HC), within one year after the first study blood draw. It is an observational, prospective study (*n* = 99,480).

These two studies are based on the development and performance evaluation of several tests, analysing different aspects of cfDNA, including ultra-deep targeted sequencing, WGS and whole-genome bisulfite (WGBS) conversion sequencing of cfDNA, tumour DNA and white-blood cell (WBC) DNA.

The ultra-deep sequencing (60,000× raw coverage) assay comprises a 508-gene panel, which allows de novo detection of tumour-derived mutations, DNA copy number aberrations and inference of tumour mutational burden (TMB), microsatellite instability, mutational signatures and sources of somatic mutations in cfDNA [72, 73]. This approach also identified an important issue related to the correct interpretation of cfDNA analysis, the need to include the analysis of genomic DNA from normal peripheral lymphocytes to discriminate variants detected in the cfDNA, which arises due to clonal haematopoiesis (CH), including changes in *DNMT3A*, *TET2*, *PPM1D* and *TP53*, in which mutations increase as a factor of age [74].

Another important aspect analysed in the CCGA trial is the development and application of genome-wide methylation profiles to identify the tissue of origin of a tumour and to aid in cancer diagnosis. In this regard, a 30× (WGBS) assay covering around 30 million CpGs across the genome was used to train a ML algorithm using data from 811 cancer methylomes (655 paraffin-embedded tissues and 156 cells isolated from tumours) to generate two tissue-of-origin models, one with mutation and copy number variant (CNV) data and the other adding methylation data. Inclusion of the methylation data improved the correct assignment of the tissue of origin in multiple cancer types [75]. This approach was also used to evaluate the prognostic implications of the detection of methylation signals associated with cancer detected in cfDNA, using WGBS using longitudinal follow-up data and indicating that tumours detected through the WGBS model in cfDNA had a worse prognosis than those without the WGBS signal [76].

To develop and test models to identify cancer using cfDNA analysis, a study including 878 cases, 580 controls and 169 assay controls (*n* = 1627) across 20 tumour types and all clinical stages, used a combination of data derived from paired cfDNA and WBC-targeted sequencing (60,000x, 507 gene panel); paired cfDNA and WBC (WGS, 35X); cfDNA (WGBS, 34X) to generate a ‘cancer-like’ signal in the cancer cases compared with non-cancer. This multi-assay modelling scheme produced a consistent ‘cancer-like’ signal, which showed an increasing trend in its detection associated with the disease stage. Interestingly, a consistent cancer signal was detected in >1% (8/580) of the non-cancer cases, a finding that the authors suggest might represent potentially undiagnosed cancer cases, this was demonstrated with a *post hoc*, follow-up analysis, during this follow-up, one of these cases was diagnosed with stage- III ovarian cancer 2 months post enrollment, other with stage-III clear-cell endometrial carcinoma 3 months post enrollment and the third participant was diagnosed with stage-IV LC 15 months post enrollment [77, 78]. This study identified that cfDNA analysis in LC can aid in the detection of early-stage LC and that the sensitivity of the analysis is comparable across histological subtypes. Of particular importance is that this study identified a significant number of variants and CNV aberrations (>50%) detected by WGS and targeted sequencing as derived from CH [79].

The point of CH of indeterminate potential (CHIP) was originally defined as the presence of cancer-related somatic mutations in WBC or marrow but without the presence of other diagnostic criteria for CH [80]. Depending on the depth sequencing, its prevalence has been reported between 2 and 33% of the cases, increasing with age [81, 82]. Its presence has also been associated with the risk for haematological neoplasms, cardiovascular diseases and overall mortality [83], and its prevalence has also been found elevated in patients with solid tumours [84]. DNA from the WBC can also contribute to the cfDNA fraction, so clonal haematopoiesis represents a source of false-negative mutations, which are not present in the tumour, but occur due to CHIP [85–87]. The ultra-deep comparison between cfDNA and WBC DNA approach used in the CCGA trial has allowed a better detection of CHIP-associated variants (91% of variants with a variant allele fraction >1%). It has also provided important information regarding its prevalence, identifying that its distribution is not significantly different in patients with cancer and non-cancer controls, and that CHIP increases with age, with an estimated 160–170% increase in CHIP-related variants per decade. Most of these variants (94%) were only present in a single individual, indicating that cfDNA analysis must be accompanied by a matched analysis of each patient WBC DNA to minimise false positives. Regarding the potential biological role of CHIP, the study also identified evidence for a strong positive selection in 21 driver genes showing CHIP-related variants. However, the cellular and biological consequences of these alterations are still not clear [88]. Recently, the results of substudies (from NCT02889978 and NCT03085888) have been published, confirming that cfDNA sequencing and the analysis of methylation patterns can detect a broad range of cancer types at metastatic and non-metastatic stages with specificity and sensitivity performance that approaches the goal of population-level screening in more than 50 cancer types, which represent 63% of all estimated cancer deaths. In cases where this approach was not able to detect cancer, tumours had a significantly better prognosis than cases detected by the tool [89, 90]. Very recently, the National Health Service (NHS) in England started a trial involving GRAIL’s Galleri^tm^ test, aiming to recruit 140,000 volunteers to evaluate its impact on early cancer detection (https://www.england.nhs.uk/2021/09/nhs-launches-world-first-trial-for-new-cancer-test/).

## Conclusions and future directions

The analysis of cfDNA is a rapidly evolving area, both regarding the methodologies used to analyse genomic alterations and in the different applications where this information might be of clinical relevance. However, there are still several points that must be addressed before cfDNA enters routing clinical application like sample-collection method (differences in blood-collection tubes: EDTA, tubes with stabilisers), centrifugation protocols and the methods for cfDNA isolation and quantification will influence the yield and quality of the cfDNA (8), the methodology selection to identify genomic alterations or specific mutations, NGS-based approaches with gene panels, whole exome or even whole-genome analysis. Even with these situations, the number of clinical trials using a cfDNA analysis approach has been steadily growing in the past ten years, and the type of genomic information, which can be obtained through its analysis, has evolved from single, specific mutations to whole exome or genome mutational analysis, resulting in the evaluation of other clinically relevant layers of information, like TMB, CNV aberration inference of mutational signatures in cfDNA. Moreover, DNA methylation patterns are becoming a powerful tool to extend the application of cfDNA analysis as a cancer-screening and early detection tool.

**Fig. 4:**
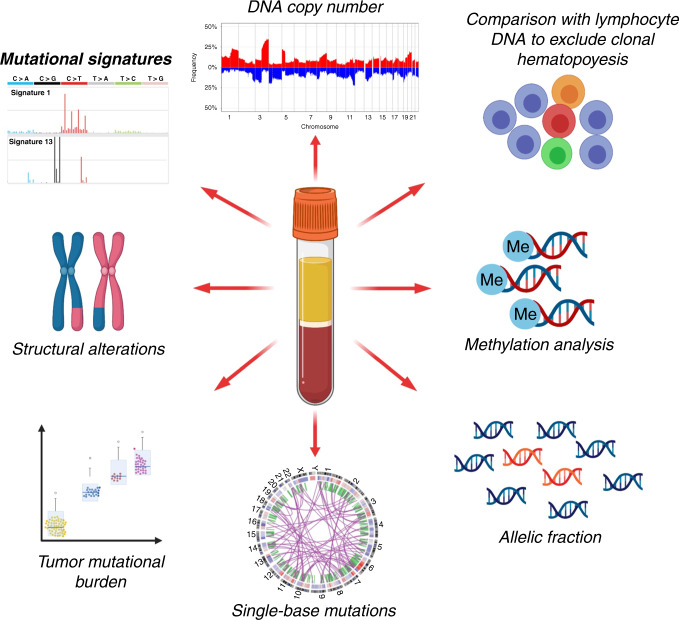
Current applications of cfDNA analysis. Current methods for cfDNA analysis go far beyond single-nucleotide substitution mutations, including detection of DNA copy number aberrations, analysis of mutational signatures, detection of chromosomal translocations, calculation of tumour mutational burden, evaluation of tumour heterogeneity through allelic fraction calculation, analysis of methylation patterns and comparison against normal DNA to detect and filter alterations due to clonal haematopoiesis.

Clinical trials constitute the closest application of cfDNA analysis in the clinical setting. As described in this review, the potential range of applications of this analysis is rapidly increasing, changing the focus from the detection of a specific set of well-characterised mutations with clinical actionability, to the characterisation of the genomic landscape of a tumour or metastatic lesion from cfDNA. The combination of different layers of information, like data derived from ultra-deep-sequencing mutational analysis, copy number aberrations and methylation patterns (Fig. 4), is opening the possibility of using cfDNA as a powerful tool not only to provide better patient follow-up and real-time treatment-response monitoring, but as a much-needed approach to improve cancer screening and early diagnosis.
